# The Story of the Insane from Year to Year

**Published:** 1902-01-25

**Authors:** 


### THE STORY OF THE INSANE FROM YEAR TO YEAR.

(Thirteenth Series.)

### GLOUCESTER COUNTY ASYLUMS.

There were 1,084 patients in residence on January 1st, 1899, and on December 31st the number had fallen to 1,067. There were 244 first admissions, 48 readmissions, 105 recoveries, 25 discharged relieved, and 115 deaths. The average number resident during the year was 1,066, and the total number of persons under care was 1,376. Calculated on the average number resident the recoveries stand at 48·94 per cent., and calculated on the average number resident the deaths stand at 10·78 per cent. Of the year's deaths 38 were caused by cerebral and spinal diseases, 20 by consumption alone or with other causes, and 11 by bronchitis with other causes. Of the year's admissions 54 owed their insanity to moral causes, 28 to hereditary influence, and 17 to intemperance in drink. Dr. Craddock's report is an extremely able and interesting one. In discussing the question of the increase of insanity and the burden on the rates thereby caused, he expresses fears, only too well grounded, that these burdens will become too grievous to be borne, and that the ratepayers "will demand the enforced application of remedies which nowadays might be regarded as beyond the range of practical politics;" and he graphically points out that "society declines to interfere with the existing state of things; that it shrinks from the endeavour to attack the evil at its source, and to interpose some obstacle to the continued intermingling of a weakly, immature, tainted and even diseased strain, with the blood of that healthy vigorous manhood, and more especially womanhood, which has gone so far to win and keep our country's high place among the rulers of mankind." Dr. Craddock is right when he speaks of marriages where it was known that the husband or wife was insane, and he does not hesitate to stigmatise these marriages as criminal, in which we fully agree with him, and we wish we could see any hope of this question being taken up by our Government; but also we fear the State is timid in asserting its rights when it should be bold, and bold in asserting them when individualism would be a better national policy. We wish Dr. Craddock success in his crusade, and regret that his platform is not wide enough to include Westminster. The Gloucestershire Asylum is one where the accommodation is in excess of the present wants of the county, and many out-county and private patients are taken in. From these large so-called profits accrue, because such out-county patients are charged both lodging-money and maintenance, whereas the Gloucestershire patients pay for the latter only. The Inland Revenue Authorities wanted to charge income-tax on these so-called profits, but such charge was to Dr. Craddock's mind (and our own too) "obviously inequitable." A long correspondence ensued, and in the end the Inland Revenue gave in and abandoned their claim, "being apparently of opinion that your committee were not assessable under Schedule D. This decision of the Inland Revenue Board was a sensible one to come to; but something should be done to prevent these unwarrantable claims being made by the local representatives of the board. A similar claim was made some years ago on the Three Counties Asylum. In this instance the claim was argued before the proper authorities, and the asylum was victorious. We have gone somewhat fully into this incident, because the matter is important in many asylums. The committee say the asylum has been maintained in good order "under the able management of Mr. Craddock." The weekly charge per head to the Gloucestershire Unions was 8s. 4d.; out-county patients pay from 13s. to 20s., and private patients 15s.

